# Power and velocity performance of swing movement in the adolescent male volleyball players – age and positional difference

**DOI:** 10.1186/s13102-024-00898-2

**Published:** 2024-05-16

**Authors:** Junsheng Wang, Zhikai Qin, Zhifeng Wei

**Affiliations:** 1 Capital University of Physical Education and Sports, Beijing, 100191 China; 2 China Volleyball College, Beijing Sport University, Beijing, 100084 China

**Keywords:** Power, Velocity, Swing movement, Spike, Serve, Adolescent male volleyball players

## Abstract

**Purpose:**

The performance of swing movement during spikes and serves plays a crucial role in determining the outcomes of volleyball matches. This study aims to explore the effects of the participation of the trunk and lower limbs’ involvement on the velocity and power of the swing movement of adolescent male volleyball players, as well as the differences in power and velocity performance of the swing movement among different ages and specific positions.

**Methods:**

The study involved 22 adolescent male volleyball players, with 11 high school students and 11 middle school students. The Kineo Globus equipment was used to assess the swing movement performance involving different segments, including arm swing movement only involving arm limb participation; upper swing movement involving trunk and arm limb participation; and whole body swing movement involving lower limb, trunk, and arm limb participation. The measured parameters included power and velocity performance levels. Before the test, each subject practiced three movement patterns twice.

**Results:**

The study found that swing movement involving both the trunk and arm limbs had significantly higher average (F = 17.70, *p* < 0.001) and peak power performance (F = 31.47, *p* < 0.001), as well as in average (F = 9.14, *p* = 0.03) and peak velocity performance (F = 23.17, *p* < 0.001). There were no significant differences in average (F = 17.70; *p* = 0.46) and peak power (F = 31.47, *p* = 0.94), as well as in average (F = 9.14, *p* = 0.99) and peak velocity performance (F = 23.17, *p* = 0.90) between movements involving the whole body and those involving the trunk and upper limbs. Among different age groups, the swing movement performance of middle school athletes showed significant enhancements in both average (F = 9.20, *p* < 0.001) and peak power (F = 19.93, *p* < 0.001), as well as in average (F = 10.75, *p* < 0.001) and peak velocity (F = 34.35, *p* < 0.001) when arm swing with trunk involvement was compared to arm swing movement. High school athletes also showed significant improvements in peak velocity (F = 34.35, *p* < 0.001), peak power (F = 17.31, *p* < 0.001), and average power (F = 9.41, *p* < 0.001) during upper swing movements, except for average velocity performance (F = 1.56, *p* = 0.21), when compared to arm swing movement. The increase rate in average velocity performance of swing movements involving trunk participation was significantly higher in middle school athletes than in high school athletes (*p* < 0.001). Among athletes in specific positions, Middle Blocker (MB) players exhibited significantly better average power performance in swing movements involving trunk and arm limb participation compared to Outside Hitter (OH) players (*p* = 0.04). Furthermore, the rate of average (*p* = 0.01) and peak (*p* = 0.03) power change during upper swing movements involving lower limb participation was significantly higher among OH players than MB players.

**Conclusions:**

The involvement of the trunk segment has been observed to significantly improve power and velocity in swing movements during spike and serves among adolescent male volleyball players. This underscores the importance of coordination between the trunk and arm in influencing swing movement performance during spikes and serves. High school athletes demonstrate superior power and velocity in arm swing movements compared to middle school athletes. MB exhibits greater power in upper limb swing movements than OH, although OH players show better coordination between the arm, trunk, and lower limb segments in the swing movement. To enhance swing movement performance in adolescent male volleyball players, particularly focusing on the trunk segment was crucial. Specialized physical training programs should target improving both arm strength and rotational power of the trunk simultaneously. This approach would help in consistently enhancing coordination between the trunk and arms, ultimately leading to optimized force generation during swing movements such as spikes and serves.

## Background

In the domain of volleyball, the spike and serve techniques are crucial for male players competing at a high level, as they greatly influence the results of matches [1–3]. Research by Oliveira et al. [4] conducted a study indicating that ball speed correlates with the velocity variables of the spike arm and trunk in volleyball players. Additionally, proper arm swing post-spike can enhance balance and lower the chances of non-contact anterior cruciate ligament injuries [5, 6]. Successful teams consistently outperform their opponents in spike and serve performance [7, 8]. Factors like athlete height, vertical jump height, and striking power have been identified as influential in both spike and serve performance. These factors have been found to exhibit a significant positive correlation with spike performance [9]. In terms of serving performance, various factors including the serve zone, serve type, the in-game role of the server, reception zone, and the receiving player are predictors of serving efficiency [10]. The influence of serve type on serve performance was the research focus. As male athletes get older, the jump serve becomes the dominant serve type. Among adolescent male volleyball players, the jump serve outperforms the standing serve across all age groups. Moreover, the power of swing movement plays a crucial role in determining the effectiveness of the jump serve [11].

The assessment of technical performance in volleyball includes various measures such as standing spikes, net spikes, general spikes, diagonal spikes in specific situations, line spikes in specific situations, and serving ball velocity. Palao and Valades [12] employed various measures to assess the specialized strength and power of the upper limbs in volleyball players. While the use of medicine ball throwing was common for evaluating upper limb power, Valade [13] suggested that improvements in medicine ball throwing distance did not have a significant impact on ball velocity performance during standing and jump spiking. This discrepancy could be attributed to the different movement patterns involved in throwing as compared to the swinging movements of spiking and serving. An emerging assessment technique is the swing kinematic chain test, which examines the contributions of the arm, trunk, and lower limb segments to the swinging motion. This test evaluates the coordination and overall performance of these segments through assessments like Arm Swing, Upper Limb Swing (trunk + arm), and Whole-body Swing (trunk + arm + legs) [14].

Additionally, Sattler et al. [15] investigated the impact of player positions on athletic performance, noting significant differences in vertical jump height among male athletes, specifically receivers and setters, but not among female players. Regarding upper limb performance, a study comparing the isokinetic performance of shoulder external and internal rotators among professional volleyball athletes in various positions. Their results indicated that attackers had significantly lower ratios of external rotation to internal rotation compared to blockers, setters, and liberos, irrespective of the rotational speed [16]. In a separate study, Milić et al. [17] found that middle blockers generally had greater height and ectomorphy, along with lower levels of mesomorphy and endomorphy compared to players in other positions. On the other hand, liberos were typically shorter, less ectomorphic, and displayed higher levels of mesomorphy and endomorphy. Additionally, Pocek et al. [18] identified significant variations in spike and block jump values across different volleyball positions, underscoring the importance of relative vertical jumping ability irrespective of position, with absolute jump values serving as a distinguishing factor not only between positions but also performance levels. In a separate study on age-related differences in athletic performance, Katia et al. [19] observed that aging alone did not lead to significant improvements in both loaded and unloaded vertical jump performances across different age groups of elite volleyball players.

Factors influencing spike and serve performance in volleyball include athlete height, vertical jump height, and striking power. Various measures have been utilized to assess the upper limb strength and power of volleyball athletes in technical performance evaluations. Swing performance has been recognized as a crucial determinant of match results. Nevertheless, there is a significant gap in research concerning swing performance across various positions and age groups among volleyball players.

## Methods

### Participants

Before the 14th Beijing City Games in 2022, we conducted tests on 22 adolescent male volleyball players from Beijing 101 Middle School’s male volleyball team. The athletes’ ages ranged from 14 to 16 years old, with 11 athletes in each of the high school and middle school groups. Table 1 displays detailed information regarding the ages, body weights, and heights of the athletes who participated in the 14th Beijing City Games, where both the high school and middle school teams achieved victory, showcasing their eligibility as elite volleyball players in China. Before the assessments, parental consent was obtained and parents were informed about the study’s objectives and possible risks. This research was approved by the Ethics Committee of the Capital University of Physical Education and Sports (Registration No. 2021A45).

**Table 1:** Basic Characteristics of Adolescent Male Volleyball Players

	High school group (*N* = 11)	Middle school group (*N* = 11)
Age (y)	16.00 ± 0.90^**^	14.55 ± 0.52^**^
Body height (cm)	189.27 ± 7.66	185.82 ± 5.36
Body mass (kg)	72.91 ± 7.73	74.36 ± 11.71

*p<0.05.

**p<0.01.

### Test procedures

The testing was conducted at the Performance Laboratory of Capital University of Physical Education and Sports, from 9:00 AM to 4:00 PM, over a single day. The laboratory maintained a temperature range of 22 to 24 °C. On the day before the tests, the team had a rest day without any physical training. Before testing, all participants underwent a 15-minute warm-up session in the physical training room. The warm-up routine consisted of (1) 5 min of jogging; (2) dynamic stretching, with a focus on the arm, shoulder, chest, and back muscle groups; (3) elastic band exercises, mimicking the arm swing, upper limb swing, and full-body swing movements performed during the tests.

Testing of Spike and Serve power and velocity performance: The power and velocity performance of the Arm swing, Upper body swin*g* (arm + trunk), and Whole-body swin*g* movements were assessed using the Kineo Globus Intelligent Resistance Strength Testing Trainer. Testing loads included 2 kg, 5% of body weight (BW), and 10% BW, to allow for comparison across different loads [14]. Pre-testing results demonstrated high test-retest reliability at these loads (*R* = 0.913). Following the methodology of Borms et al. [20], participants were instructed to hold a 2 kg medicine ball with both hands, arms extended, and elbows flexed at 90 degrees. They were then instructed to throw the medicine ball forward while maintaining contact of the head, shoulders, and back with the wall. Consistent with prior studies, the tests included 2 kg resistance arm swing, 5% BW resistance arm swing, and 10% BW resistance arm swing protocols [21, 22]. The testing sequence (arm swing, upper body swing, whole-body swing) was standardized.

**Fig. 1:**
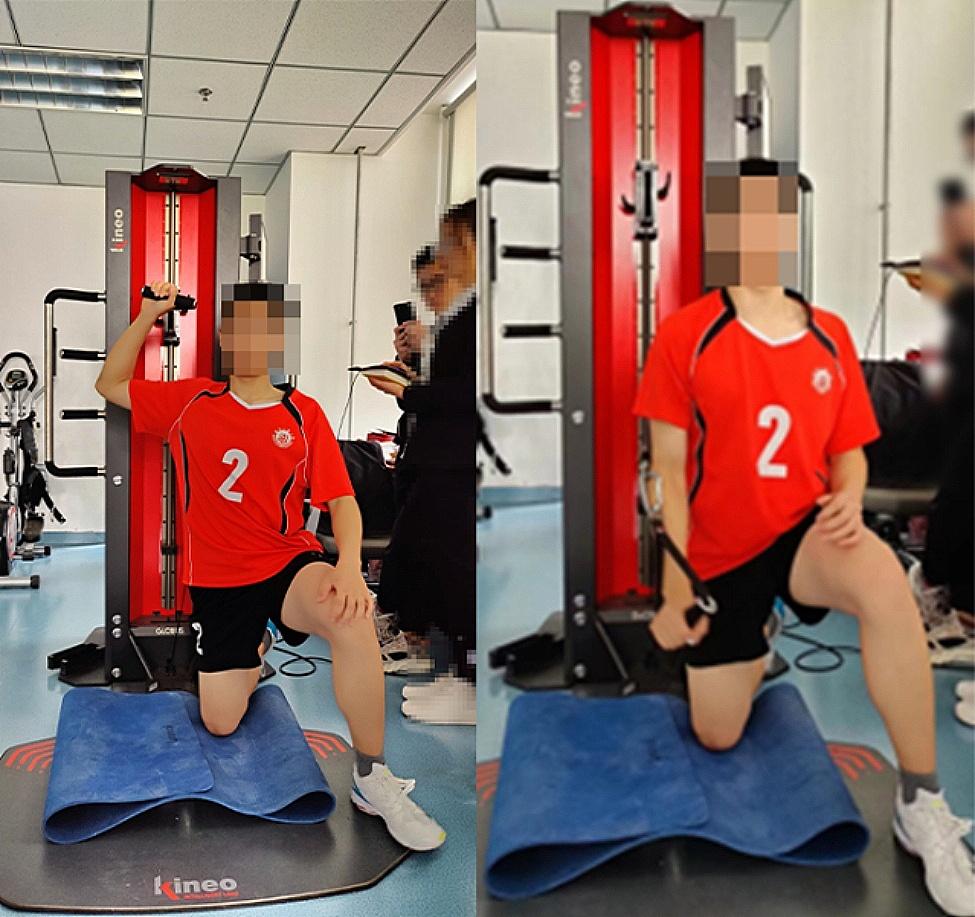
Arm swing movement

Regarding the requirements for the test movements:

(1) During the execution of the arm swing movement, athletes were required to assume a kneeling position with one knee on the ground, the trunk fixed forward and constantly perpendicular to the ground, with only the arm segment participating in the swing movement. Figure 1 below.

In the context of a right-handed spike, the left leg was positioned in front with the knee bent at a 90-degree angle. The trunk and hip joints remained fixed, ensuring they were perpendicular to the ground. A long rod was placed behind the torso as a reference point to prevent compensatory force generation by the athlete’s trunk, enabling only the arm to execute the swing movement.

**Fig. 2:**
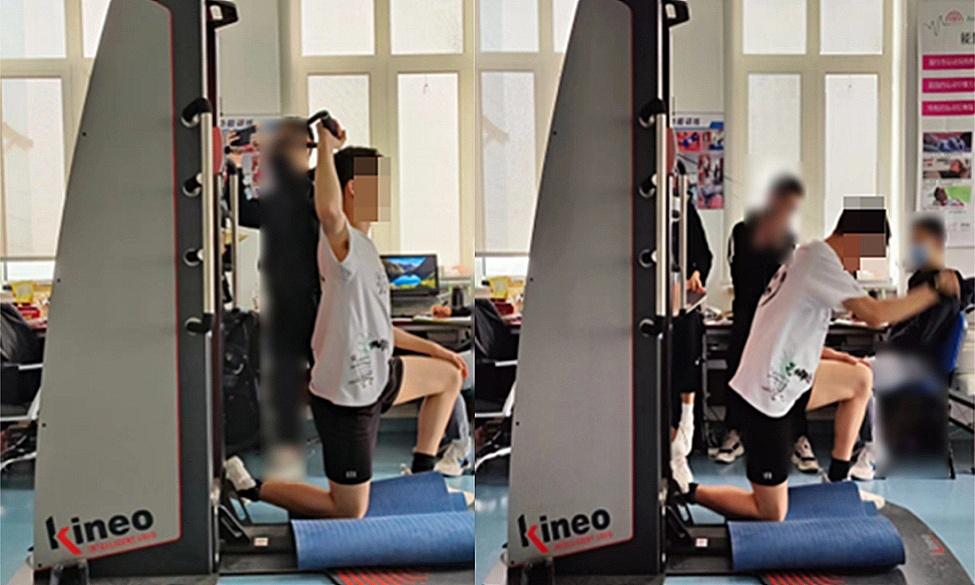
Upper body swing movement

(2) For the upper body swin*g*, athletes were required to kneel with one knee on the ground, the lower limbs immobilized, and both the trunk and arm segments participated in the swing movement. Figure 2 below.

When performing a kneeling position with one knee down, like with the right-hand spike, the starting position requires the left leg to be positioned in front with the knee joint at a 90-degree angle and the hip joint perpendicular to the ground. Following this, the trunk should be rotated and the arms pulled back. Athletes were then instructed to keep the lower body still below the hip joint while rotating the upper body above the navel and swinging the arm in a swing movement. This exercise incorporates trunk extension, flexion, and rotation to execute the upper-body swing.

**Fig. 3:**
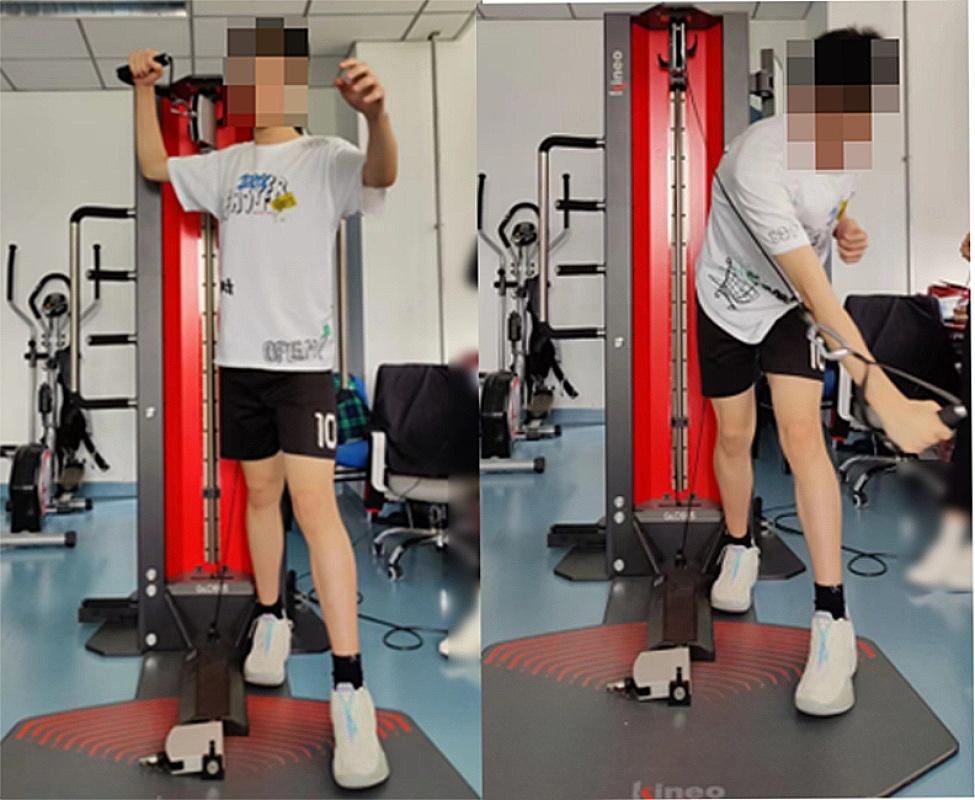
Whole-body swing movement

(3) When performing the whole-body swin*g*, athletes were required to stand with legs apart (left foot in front), with all lower limbs, the trunk, and the arm segment collectively participating in the swin*g* movement. The test metrics included average power, peak power, average velocity, and peak velocity. Figure 3 below.

Standing position, taking a right-handed spike as an example, with the left leg forward and the left shoulder facing the direction of the swinging arm, resembling a volleyball serve or an in-place spike movement. At the end position, relying on pushing off the ground with the lower limbs, hip rotation, body rotation, and arm swing, smoothly complete the full spiking arm movement.

Participants were informed of the testing requirements for the movements one week prior, and elastic bands were used during regular training sessions to simulate the test movements. Before the official testing, participants were given two practice opportunities to ensure the accuracy of their movements during the formal test. During the formal testing, each test movement was repeated three times, with a 30-second rest interval between repetitions and a 3-minute rest interval between sets. The entire formal testing process was recorded on video for later analysis.

### Statistics analysis

Video recordings were analyzed to select the best-executed and most technically sound repetitions from the three test trials for analysis. All variables were normally distributed and represented using mean ± standard deviation. The Kolmogorov-Smirnov test was analyzed, where the metrics were found to follow a normal distribution. Differences in power and velocity performance among athletes in arm swing, upper body swing (arm + trunk), and whole-body swing(trunk + arm + legs)movement performance were analyzed using a ONE-WAY ANOVA. Independent samples t-tests were used to analyze differences in power and velocity performance between athletes of different ages and specialized positions. Statistical significance was considered present when p-value < 0.05 in all data comparisons. To reduce the risk of type I error inflation, researchers frequently utilize Bonferroni correction. If the calculated p-value falls below 0.01 (**: *p* < 0.01; *p < 0.05), the results were considered statistically significant.

## Results

**Fig. 4:**
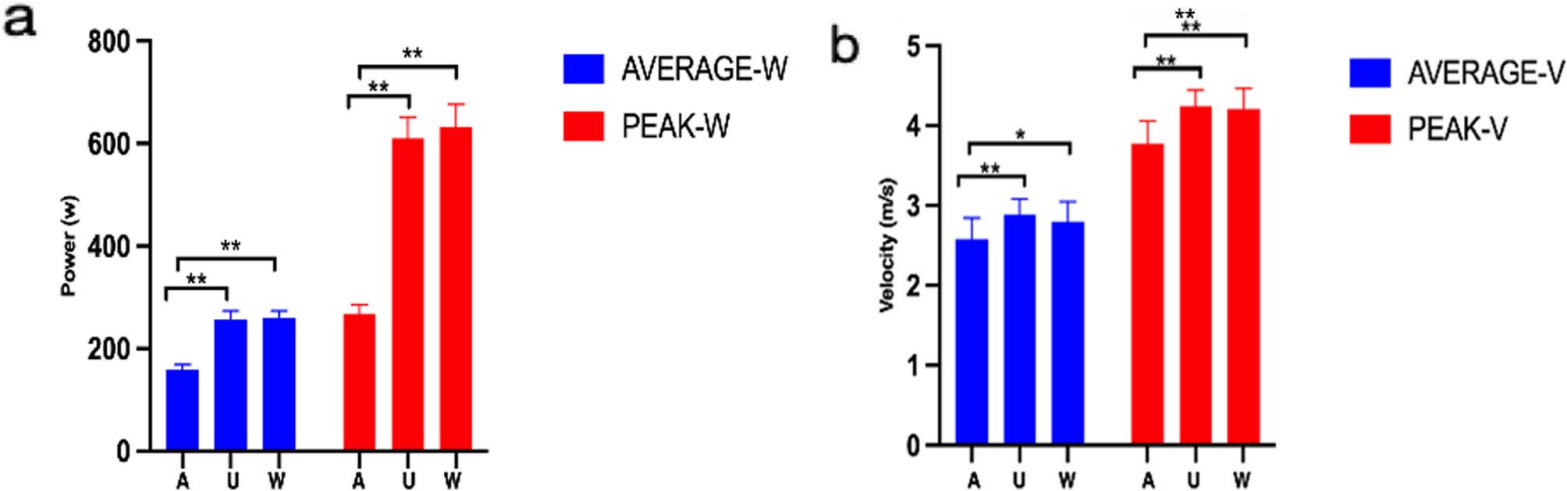
Power and velocity performance in swing movements with different limb involvements. (**a** The difference in power of different parts involved in arm swing. **b** The difference in velocity of different parts involved in lower arm swing. A means arm swing; U means upper Limb; W means whole body. All data were expressed as Mean ± SD). *p<0.05, **p < 0.01

Tables 2 and 3; Fig. 4 present the results. When comparing the upper body swing movement with trunk participation to the arm swing movement, there were significant improvements in average (F = 17.70, *p* < 0.001) and peak power performance (F = 31.47, *p* < 0.001), as well as in average (F = 9.14, *p* < 0.001) and peak velocity performance (F = 23.17, *p* < 0.001). The average power increased by 68.36% and the peak power increased by 135.2%. Additionally, the average velocity increased by 12.33%, and the peak velocity increased by 12.57%. On the other hand, the introduction of lower limb involvement in the whole-body swing movement did not result in significant changes in average (F = 17.70; *p* = 0.46) and peak power (F = 31.47, *p* = 0.94), as well as in average (F = 9.14, *p* = 0.99) and peak velocity performance (F = 23.17, *p* = 0.90), and in some cases, even exhibited a decrease. The rate of change (ROC%) in swing movement showed that trunk and lower limb involvement enhanced the performance. Comparing the rate of power change (A-UAW-ROC/A-UPW-ROC) and the rate of velocity change (A-UAV-ROC/A-UPV-ROC), it was found that trunk participation had significantly higher values than lower limb involvement (*p* < 0.01).

**Table 2:** Swing movement velocity performance

	Total	High	Middle	OH	MB	OP
Arm AV (m/s)	2.58 ± 0.262	2.71 ± 0.23*	2.45 ± 0.23*	2.60 ± 0.26	2.70 ± 0.29	2.42 ± 0.30
Arm PV (m/s)	3.78 ± 0.29	3.90 ± 0.29*	3.66 ± 0.23*	3.79 ± 0.22	3.78 ± 39.72	3.81 ± 23.29
Upper AV (m/s)	2.88 ± 0.20	2.88 ± 0.16	2.88 ± 0.5	2.78 ± 0.15	2.98 ± 0.18	2.84 ± 0.23
Upper PV (m/s)	4.24 ± 0.21	4.26 ± 0.26	4.21 ± 0.14	4.21 ± 0.40	4.25 ± 0.20	4.25 ± 0.06
Whole Body AV (m/s)	2.80 ± 0.25	2.77 ± 0.29	2.82 ± 0.22	2.84 ± 0.23	2.70 ± 0.33	2.72 ± 0.23
Whole Body PV (m/s)	4.21 ± 0.26	4.26 ± 0.34	4.16 ± 0.14	4.22 ± 0.12	4.07 ± 0.44	4.26 ± 0.11
A-UAV-ROC (%)	12.33 ± 9.54	6.80 ± 7.07*	17.86 ± 8.59*	7.00 ± 6.04	11.00 ± 10.75	18.20 ± 9.39
A-UPV-ROC (%)	12.57 ± 7.33	9.63 ± 8.28	15.50 ± 5.04	11.40 ± 7.96	13.00 ± 8.25	11.80 ± 5.85
U-WAV-ROC (%)	2.74 ± 9.38	-3.76 ± 9.19	-1.71 ± 9.90	2.80 ± 9.12	-9.33 ± 10.69	-4.00 ± 9.41
U-WPV-ROC (%)	-0.54 ± 5.65	0.10 ± 7.45	-1.18 ± 3.25	0.80 ± 6.61	-4.33 ± 7.69	0.20 ± 1.79

Total: All athletes; High: High school group; Middle: Middle school group; OH: Outside Hitter; MB: Middle Blocker; OP: Opposite Hitter; AV: Average Velocity; PV: Peak Velocity; A-UAV ROC = (Upper AV-Arm AV) × 100%; A-UPV-ROC = (Upper PV-Arm PV) × 100%; U-WAV-ROC = (Whole Body AV-Upper AV) × 100%; U-WPV-ROC = (Whole Body PV-Upper PV) × 100%. * Independent samples t-tests revealed a statistically significant difference between groups (*p* < 0.05).

**Fig. 5:**
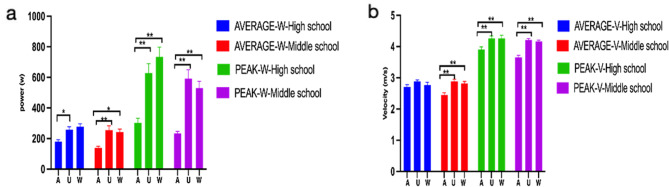
Power and velocity performance in swing movement with different limb involvements for high school and middle school athletes. (**a** Differences in the power of different parts of the lower swing arm between high school and middle school athletes. **b** Differences in velocity of different parts of the lower swing arm between high school and junior middle school athletes. A means arm swing; U means upper Limb; W means whole body. All data were expressed as Mean ± SD). *p < 0.05, **p < 0.01

In different age groups, the swing movement performance of middle school athletes showed significant enhancements in both average (F = 9.20, *p* < 0.001) and peak power (F = 19.93, *p* < 0.001), as well as in average (F = 10.75, *p* < 0.001) and peak velocity (F = 34.35, *p* < 0.001) when arm swing with trunk involvement was compared to arm swing movement (see Tables 2 and 3; Fig. 5). High school athletes also exhibited significant improvements in peak velocity (F = 34.35, *p* < 0.001), peak power (F = 17.31, *p* < 0.001), and average power (F = 9.41, *p* < 0.001) in upper swing movement, except for average velocity performance (F = 1.56, *p* = 0.21), when compared to arm swing movement. Furthermore, there were no significant changes observed in average (F = 10.75, *p* = 0.80) and peak velocity (F = 34.35, *p* = 0.77), as well as in power average (F = 9.20, *p* = 0.90) and peak power (F = 19.93, *p* = 0.57) performance in swing movements with lower limb involvement compared to upper swing movement in middle school athletes. Among high school athletes, both average (F = 1.56, *p* = 0.52) and peak (F = 5.34, *p* > 0.99) velocities, as well as average (F = 9.41, *p* = 0.70) and peak (F = 17.31, *p* = 0.36) power, showed similar results.

An investigation was conducted to compare swing movement performance in male volleyball athletes across different age groups. The study focused on middle school and high school athletes, analyzing varying levels of limb involvement. Results from Tables 2 and 3 indicated that high school athletes exhibited superior average (*p* = 0.02) and peak (*p* = 0.04) velocity, as well as in average (*p* = 0.02) and peak (*p* = 0.05) power in arm swing movement when compared to middle school athletes. In terms of the rate of change in swing movement performance with different limb involvements, middle school athletes showed a notably higher growth rate in average velocity performance of swing movements involving trunk participation compared to high school athletes (*p* < 0.001). This suggests a better coordination between trunk and arm segments among middle school athletes.

**Table 3:** Swing movement power performance

	Total	High	Middle	OH	MB	OP
Arm AW (w)	160.00 ± 43.10	180.27 ± 42.44*	139.55 ± 34.52*	159.0 ± 30.36	184.5 ± 44.85	150.6 ± 59.45
Arm PW (w)	269.00 ± 83.40	303.00 ± 100.29*	234.00 ± 43.97*	250.6 ± 70.81	314.7 ± 116.2	247.4 ± 47.25
Upper AW (w)	257.00 ± 77.50	258.64 ± 62.00	255.27 ± 93.62	203.2 ± 46.49*	313.2 ± 95.61*	234.0 ± 49.38
Upper PW (w)	610.00 ± 195.0	628.00 ± 204.58	592.00 ± 194.01	463.6 ± 241.7	683.7 ± 246.4	634.0 ± 145.2
Whole Body AW (w)	260.00 ± 64.90	277.73 ± 60.87	242.18 ± 66.70	261.8 ± 56.24	250.0 ± 72.69	230.4 ± 72.69
Whole Body PW (w)	633.00 ± 205.0	734.55 ± 210.85*	530.45 ± 145.19*	639.8 ± 175.9	519.2 ± 269.0	660.4 ± 178.8
A-UAW-ROC (%)	68.36 ± 63.68	45.61 ± 26.76	91.12 ± 81.61	29.20 ± 26.02	83.5 ± 101.90	66.40 ± 37.25
A-UPW-ROC (%)	135.20 ± 71.06	116.13 ± 79.20	151.50 ± 62.47	87.20 ± 93.40	126.3 ± 85.38	155.8 ± 34.90
U-WAW-ROC (%)	5.17 ± 26.71	9.31 ± 21.34	1.02 ± 31.71	31.60 ± 31.65*	-17.83 ± 19.04*	-2.80 ± 12.76
U-WPW-ROC (%)	-11.45 ± 49.23	25.81 ± 58.34	-2.90 ± 35.10	60.00 ± 76.72*	-24.83 ± 26.01*	6.20 ± 24.44

Total: All players; High: High school group; Middle: Middle school group; OH: Outside Hitter; MB: Middle Blocker; OP: Opposite; AW: Average Power; PW: Peak Power; A-UAW-ROC = (Upper AW - Arm AW) × 100%; A-UOW-ROC = (Upper PW - Arm PW) × 100%; U-WAW-ROC = (Whole Body AW - Upper AW) × 100%; U-WPW-ROC = (Whole Body PW - Upper PW) × 100%. * Independent samples t-tests revealed a statistically significant difference between groups (*p* < 0.05).

**Fig. 6:**
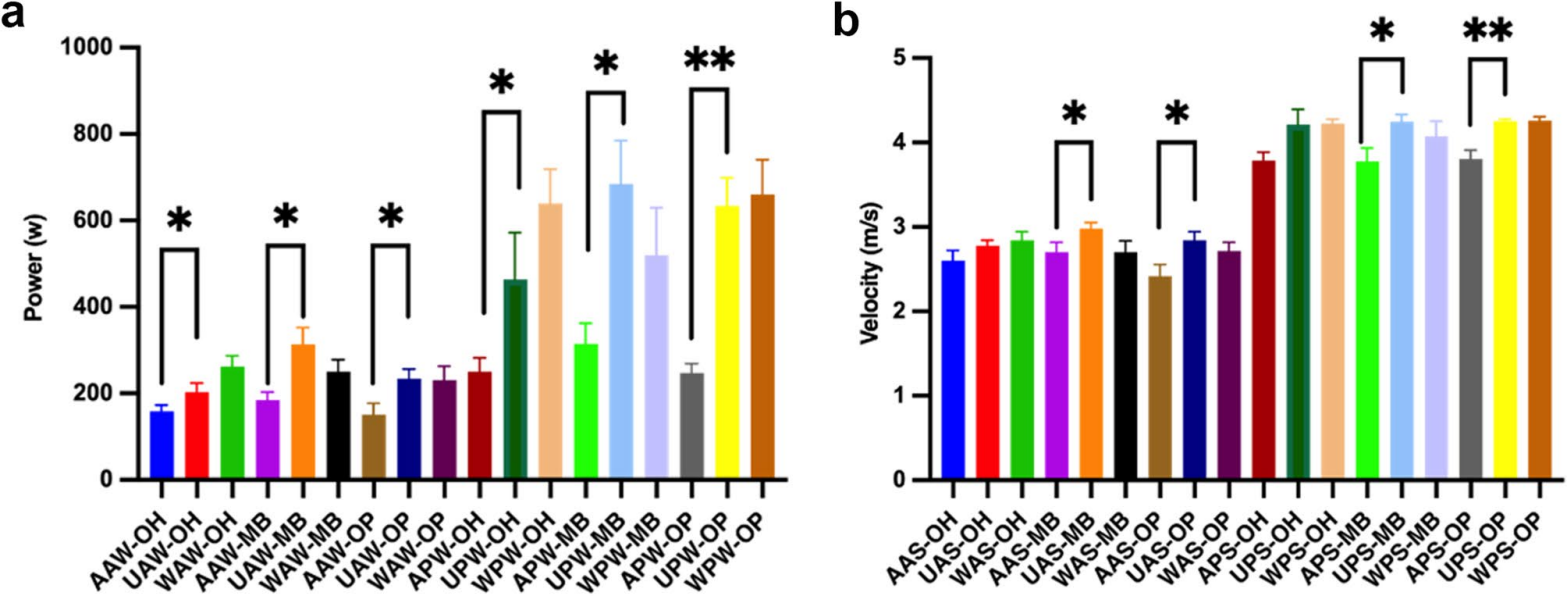
Illustrates the power and velocity performances of athletes in various positions during swing movement with different segmental involvements. (**a** The power performance of the arm swing with the participation of different parts of each specialized athlete. **b** The arm swing velocity performance of each specialized athlete with the participation of different parts. A means arm swing; U means upper Limb; W means whole body. All data were expressed as Mean ± SD). *p<0.05, **p<0.01

In Tables 2 and 3; Fig. 6, the swing movement performances of athletes in specific positions were presented. When comparing arm swing to upper limb swing movement with trunk engagement, significant enhancements in both average (F = 4.71, *p* = 0.02) and peak power (F = 4.20, *p* = 0.03) were observed for middle blockers (MB). Additionally, the average (F = 6.036, *p* = 0.05) and peak (F = 6.39, *p* = 0.01) power of outside hitters (OH), as well as the average (F = 4.55, *p* = 0.05) and peak (F = 14.50, *p* < 0.001) power of opposites (OP), all yielded similar results. In terms of velocity performance, significant improvements were observed in the arm velocity of MB and OP, while the increase in arm average (F = 1.60, *p* = 0.45) and peak (F = 4.20, *p* = 0.07) velocity for OH was not significant. However, there were no significant changes in swing power and velocity when the lower limbs were involved for all athletes. Comparing the swing movement of OH, MB, and OP, it was found that the upper limb swing average power of MB was superior to that of OH (*p* = 0.04), while there were no significant differences between OP and OH. Only OH showed a significantly superior rate of average (*p* = 0.01) and peak (*p* = 0.03) power change when the lower limbs were involved compared to MB. As shown in Fig. 6. This suggests that the coordination between the lower limbs, trunk, and arms in OH was superior to that in MB, and MB experienced a decrease in peak power when the lower limbs were involved (*p* = 0.42). Although the above conclusions were drawn, the results of this study were limited by factors such as sample size, selection bias, and potential confounding variables.

## Discussion

Among different segments involved, the findings of this study demonstrated that involving the trunk segment significantly enhanced the power and velocity performance of swing movements in adolescent male volleyball players. However, the inclusion of the lower limb segment had a limited impact on athletes’ swing movement performance. Among athletes of different age groups, high school athletes demonstrated superior power and velocity performance in arm swing movement, as well as peak power performance in whole-body swing movement compared to middle school athletes. On the other hand, middle school athletes exhibited better coordination between the arm and trunk segments. When considering player positions, MB showed superior power performance in upper swing movement compared to OH, resulting in decreased swing movement power when the lower limb was involved. However, OH players displayed better coordination among the arm, trunk, and lower limb segments during swing movement.

The involvement of the trunk segment has been shown to improve power and velocity in swing movements. However, the contribution of the lower limb segment does not appear to have a significant impact on swing performance. Yapıcı et al. [23] conducted a study that revealed that athletes in a water polo match were able to complete the shooting motion using only their upper limbs, despite immobilization of the ankles, knees, and thighs. Additionally, highlighted that shooting without lower limb involvement led to quicker completion of the shooting preparation, underscoring the importance of trunk, back, and shoulder strength for rapid shooting in water polo [24]. In water polo athletes, the flexion of the body, extension of the trunk, and flexion-extension movements of the shoulder joint were significantly correlated with shot velocity measurements [25–27]. In volleyball disciplines, serving types and spiking techniques are typically performed mid-air without the involvement of the lower limbs, except for the standing serve. Therefore, training for volleyball athletes should focus not only on enhancing the performance of the arm swing movement, but also on improving the coordination between the trunk and arm segments.

Among athletes of different age groups, high school-level athletes outperformed their middle school counterparts in arm swing, upper body swing, and whole body swing movement performance. Specifically, the arm swing movement demonstrated significant differences in power and velocity between middle and high school athletes. Guntur and Fuchs et al. [28, 29] argued that from a gender difference perspective, coordination significantly differs in spiking outcomes. Our study supplements the existing knowledge by demonstrating differences in arm swing power and velocity among athletes of different age groups. Moreover, middle school athletes exhibited better coordination between the arm and trunk segments compared to their high school counterparts in terms of movement segment coordination. In research on the differences in athletic performance among athletes of different ages, Melrose et al. [30] found significant differences between age groups of volleyball players: height, weight, lean body mass, body circumferences, isometric strength, and serving velocity were all higher in Group B (15–17 years) compared to Group A (12–14 years). As individuals aged, there was a gradual increase in both arm circumference and strength in young men, resulting in a more effective swing effect of the upper limbs. In contrast, middle school students typically had weaker upper limb strength but showed better coordination in movements like turning shoulder retraction, and rotation. Gonçalves et al. [31] suggested that elite volleyball players with several years of experience outperform sub-elite players in all upper and lower limb strength variables, with the most pronounced differences observed in the OH position. Fuchs et al. [28] proposed that enhancing arm speed through upper body momentum relies not on shoulder joint strength but on the coordination between the arms and upper body. Proper arm swing allows for the upper body to extend earlier and faster, leading to increased power generation. The results of Ferris et al. [32] on NCAA Division I volleyball players indicate that shoulder extension strength and coordination of the upper body were the main variable physiological factors related to spiking velocity. Therefore, training programs should consider an individual’s upper body strength, trunk rotation strength, and combined rotational power.

Among athletes in various specialized positions, MB demonstrated superior upper-body swing power compared to OH. However, no significant differences were found among players in other positions. OH showed more effective coordination among the arm, trunk, and lower body segments in the swing movement. This discovery is noteworthy considering Pion et al.‘s [33] emphasis on the importance of movement coordination for achieving elite performance levels. A study conducted by Viviani and Baldin compared the physique of adolescent volleyball players (under 18 years old) and senior players (18 years old and above) with that of girls of the same age who did not partake in volleyball matches. The study revealed that OH were taller, heavier, and more robust than other players [34]. Specifically, the engagement of lower body segments constrained the swing movement performance of MB, who were tasked with executing quick tactics and requiring high-velocity swing movements to enhance upper body swing power. A study by Kim et al. [16] found that attackers exhibited lower ratios of shoulder external to internal rotation in comparison to blockers, setters, and liberos, across various positions in professional volleyball. Gonçalves et al.‘s [31] study found that OP athletes performed better in a 3 kg medicine ball throw when their trunk and lower limb segments were immobilized. In trunk-involved throwing tests, there were no significant differences in throwing distance among OH, OP, and MB [35]. However, Milic et al. [17] study showed that the athletic abilities of adolescent female athletes specializing in outside hitter, setter, and opposite positions could be differentiated based on a 2 kg medicine ball throw, with no significant differences found among these specific positions. Previous research on the differences in upper limb power among athletes in different positions has yielded inconsistent results. This variation can be attributed, in part, to the use of different testing methods. For example, in medicine ball throwing tests, athletes may be seated against a wall or in supine positions to stabilize the trunk and lower limbs during the test. These variations in test movements can lead to differences in results. In this study, we aimed to further explore the performance of the swing movement involving the arm, trunk, and lower limbs. Our test movement closely resembled the spike and serve movements observed in volleyball. We assessed the power and velocity of the swing movemen*t* by considering the role of each segment in the chain of movement. This methodology could potentially be utilized to analyze the swing performance of the arms, trunk, and lower limbs in elite volleyball players of varying genders and positions. Implementing intervention strategies to improve swing performance has the potential to transform the training and selection of elite volleyball players.

## Conclusion

The involvement of the trunk segment has been noted to significantly enhance power and velocity in swing movements during spike and serves in adolescent male volleyball players, while the impact of the lower limbs appears to be less pronounced. This highlights the importance of coordination between the trunk and arm in influencing swing movement performance during spikes and serves. High school athletes outperform middle school athletes in arm swing, upper body swing, and whole-body swing movements. Specifically, power and velocity in arm swing movements were key differentiators between the two groups. MB shows greater power in upper limb swing movements compared to OH, but OH players exhibit better coordination between the arm, trunk, and lower limb segments in the swing movement chain.

To improve swing movement performance in adolescent male volleyball players, a detailed analysis of different components, especially focusing on the trunk segment, was essential. Specialized physical training programs should have targeted the enhancement of both arm strength and rotational power of the trunk simultaneously. This approach would have facilitated the consistent improvement of coordination between the trunk and arms, ultimately resulting in optimized force generation during swing movements like spikes and serves.

## Data Availability

The datasets used and/or analyzed during the current study are available from the corresponding author on reasonable request.
